# Genomics- and Transcriptomics-Guided Discovery of Clavatols from Arctic Fungi *Penicillium* sp. MYA5

**DOI:** 10.3390/md22060236

**Published:** 2024-05-22

**Authors:** Yuan-Yuan Sun, Bo Hu, Hao-Bing Yu, Jing Zhou, Xian-Chao Meng, Zhe Ning, Jin-Feng Ding, Ming-Hui Cui, Xiao-Yu Liu

**Affiliations:** 1 Naval Medical Center of PLA, Department of Marine Biomedicine and Polar Medicine, Naval Medical University, Shanghai 200433, China; sunyy3636@163.com (Y.-Y.S.); hb8601@163.com (B.H.); yuhaobing1986@126.com (H.-B.Y.); mxc0960623@163.com (X.-C.M.); ningzhe95@163.com (Z.N.); 2022220806@jou.edu.cn (J.-F.D.); 13156270892@163.com (M.-H.C.); 2 Institute of Quality Inspection and Technical Research, Shanghai 200031, China; zhoujing2@sqi.org.cn

**Keywords:** *Penicillium* sp., genomics, transcriptomics, secondary metabolite, clavatols

## Abstract

Clavatols exhibit a wide range of biological activities due to their diverse structures. A genome mining strategy identified an *A5cla* cluster from *Penicillium* sp. MYA5, derived from the Arctic plant *Dryas octopetala*, is responsible for clavatol biosynthesis. Seven clavatols, including one new clavatol derivate named penicophenone F (**1**) and six known clavatols (**2**–**7**), were isolated from *Penicillium* sp. MYA5 using a transcriptome mining strategy. These structures were elucidated by comprehensive spectroscopic analysis. Antibacterial, aldose reductase inhibition, and siderophore-producing ability assays were conducted on compounds **1**–**7**. Compounds **1** and **2** demonstrated inhibitory effects on the ALR2 enzyme with inhibition rates of 75.3% and 71.6% at a concentration of 10 μM, respectively. Compound **6** exhibited antibacterial activity against *Staphylococcus aureus* and *Escherichia coli* with MIC values of 4.0 μg/mL and 4.0 μg/mL, respectively. Additionally, compounds **1**, **5**, and **6** also showed potential iron-binding ability.

## 1. Introduction

Iron is an indispensable element for plant growth and development. Iron mainly exists in the form of insoluble substances such as iron oxide or iron hydroxide, which is difficult to be directly absorbed by plants [1]. The endophytic fungi in the vast majority of plants can synthesize siderophores, facilitating increased iron absorption by the host from the surrounding environment [2,3,4,5,6,7]. Some endophytes residing within plants cultivated in extreme environments enhanced host plant resistance to nutrient deficiencies and cold conditions by producing siderophores [8,9,10]. Siderophores, which function as iron chelators secreted by microorganisms, can be classified into four groups based on their functional groups, including catecholates, hydroxamates, carboxylates, and phenolates [11,12,13].

Clavatols, a family of aromatic polyketides with phenolic groups, play a pivotal role in microorganisms against adverse environmental changes. Clavatols showed inhibitory effects against various plant pathogenic fungi, such as *Staphylococcus greyi*, *Dictyostelium tylosporium*, and *Mycosphaerella solanacearum* [14]. Furthermore, clavatol derivatives revealed structure diversity and a variety of biological activities, such as antiviral [15] and inhibitory activity against *Mycobacterium tuberculosis* protein tyrosine phosphatase B [16].

**Figure 1:**
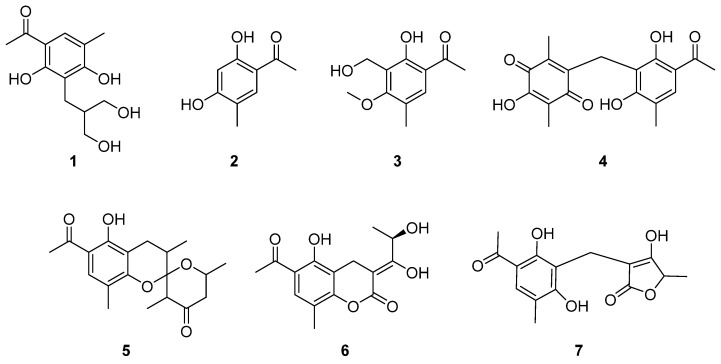
Structures of the isolated compounds **1**–**7**.

The development and popularity of sequencing technology have led to a new trend in the genome-directed discovery of specified secondary metabolites. Through our investigation focusing on siderophores, it was elucidated that *Penicillium* sp. MYA5 possesses the capacity to produce siderophores (Figure S1). Genome sequencing and mining of secondary metabolism biosynthesis gene clusters in *Penicillium* sp. MYA5 revealed gene clusters related to clavatols. Eight clavatols, including one new clavatol derivate, penicophenone F (**1**), and seven known clavatols (**2**–**7**), were isolated from *Penicillium* sp. MYA5 based on transcriptome mining strategy (Figure 1). The structures of all the compounds were unambiguously elucidated by extensive spectroscopic analysis, complemented by literature comparisons. This study contributes to the comprehensive understanding of clavatols, encompassing their discovery, isolation, purification, structural characterization, and bioactivity evaluation.

## 2. Results

### 2.1. Based on Genomics and Transcriptomics to Discovery Clavatols

The strain *Penicillium* sp. MYA5 underwent genome sequencing using the Illumina HiSeq 2000 platform. The complete genome was composed of 32,800,485 bp, with an average GC content of 47.97%. Genome annotation resulted in the detection of 11,248 protein-coding sequences, 235 tRNA genes, and 55 rRNA operons.

For the determination of the evolutionary relationship of *Penicillium* sp. MYA5 with other *Penicillium* strains, a whole-genome core SNP-based phylogenetic tree was constructed and phylogenetic analysis revealed that *Penicillium* sp. MYA5 was closely related to *Peniciilium isariiforme* (Figure S2).

Secondary metabolite biosynthetic gene clusters (BGCs) in the draft genome of *Penicillium* sp. MYA5 were predicted using the antiSMASH version 6.1.1 [17]. The results revealed that *Penicillium* sp. MYA5 could synthesize abundant secondary metabolites, which might be an important source of novel bioactive compounds. Forty-six completely sequenced biosynthetic gene clusters were predicted in the *Penicillium* sp. MYA5 genome and contained seven polyketide synthases (PKSs), twenty non-ribosomal peptide synthetases (NRPSs), twelve hybrid NRPS-PKSs, five terpenes, two indoles, and others. The details of the location of all the sequenced biosynthetic gene clusters are shown in Table S2.

**Figure 2:**
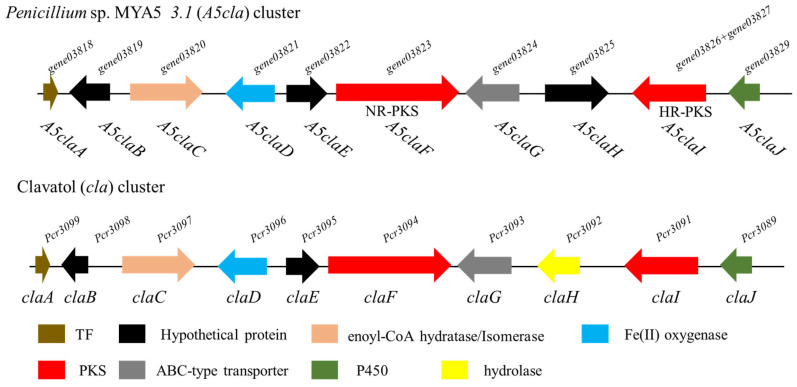
Schematic representation of cluster *3.1* (*A5cla*) and cluster *cla* in *Penicillium crustosum* [18].

Aromatic polyketones were synthesized by non-reducing polyketide synthase (NR-PKS) in fungi and the *Penicillium* sp. MYA5 genome contained three NR-PKS-containing clusters. We identified a unique NR-PKS cluster *3.1* (*A5cla*) as a candidate biosynthetic gene cluster. In order to postulate putative cluster products from the identified cluster, we conducted BLAST analyses of all the clusters across the genomes present in the GenBank archive. This identified cluster *3.1* (*A5cla*) with high homology to cluster *cla* in *Penicillium crustosum* [18] (Table S3), for which the biosynthetic product had been experimentally determined as clavatols, allowing us to predict clavatols that are produced by *Penicillium* sp. MYA5. The *A5cla* cluster comprised eleven ORFs, encoding one NR-PKS (gene03823), two HR-PKSs (gene03826 and gene03827), one Enoyl-CoA isomerase (gene03820), one cytochrome P450 oxidase (gene03828), one Fe (II) oxygenase (gene03821), one ABC-type transporter (gene03824), one transcription factor (gene03818), and three hypothetical proteins (gene03819, gene03822, and gene03825) (Figure 2).

**Figure 3:**
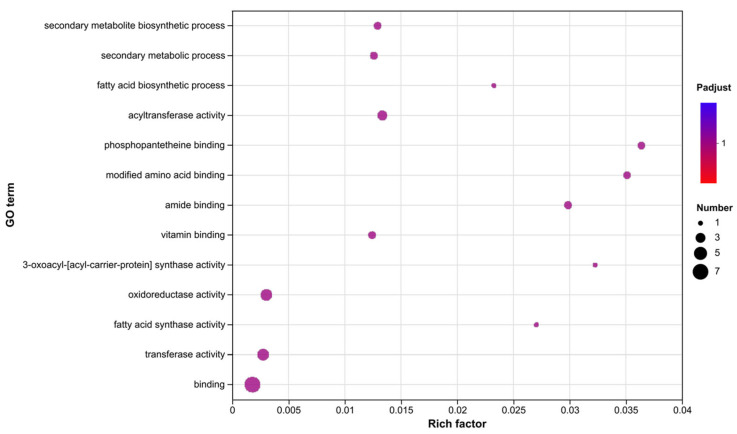
GO enrichment analysis of the cluster *A5cla*.

We performed GO enrichment analysis using *A5cla* to identify its involvement with secondary metabolic processes. We searched the genes of each GO term whose function related to transferase activity, acyltransferase activity, oxidoreductase activity, modified amino acid binding, etc. There were two genes (gene03823 and gene03827) under each of the GO terms “secondary metabolite biosynthetic process” and “secondary metabolic process” (Figure 3) and four genes (gene03821, gene03823, gene03824, and gene03828) under “oxidoreductase activity”, “transferase activity”, and “binding” (Figure 3). In this study, by annotating the gene function through GO analyses, we identified two key genes and four modification genes.

However, cluster *A5cla* still differed from the identified cluster *cla*, suggesting that further research on *Penicillium* sp. MYA5 would undoubtedly be worthwhile.

**Figure 4:**
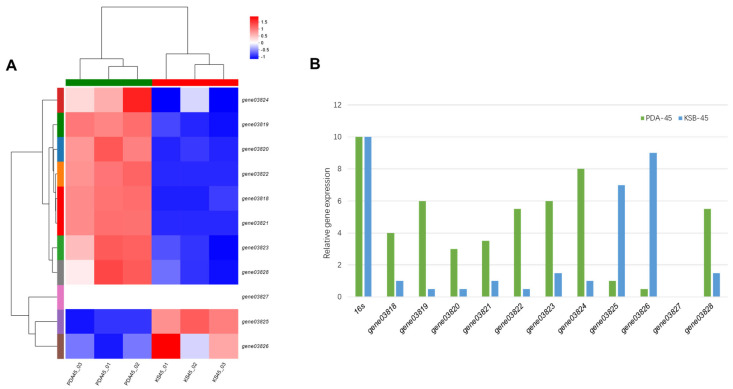
Transcriptomic analyses of cluster *A5cla* for different culture conditions. (**A**) Cluster heatmap of metabolite abundance based on hierarchical cluster analysis; (**B**) The quantitative real-time PCR (qPCR) results of several genes.

To investigate the optimum cultivation conditions of secondary metabolite accumulation in *Penicillium* sp. MYA5, we selected PDA medium for 45 days and KSB medium for 45 days of fermentation of crude extract for transcriptome analysis. The expression patterns of the eleven genes in cluster *A5cla* were analyzed using clustering analysis (Figure 4A). The figure shows that *gene03818*–*gene03824* and *gene03828* exhibited higher expression levels under PDA culture conditions for 45 days, while *gene03825* and *gene03826* demonstrated the highest expression levels under KSB culture conditions for 45 days. Additionally, quantitative real-time polymerase chain reaction (RT-qPCR) was employed to determine the expression profiles of target gene clusters under two different culture conditions. The results showed that the *A5cla* cluster exhibited higher gene expression under PDA culture conditions for 45 days compared to KSB culture conditions for 45 days (Figure 4B). The strains cultured in *Penicillium* sp. MYA5 on PDA for 45 days exhibited high expression of *gene03819*, *gene03823*, *gene03824*, and *gene03828* and low expression of *gene03825* and *gene03826* (Figure 4B). Considering the expression profiles of *A5cla* under different culture conditions, we ultimately selected the PDA culture for large-scale fermentation for 45 days. 

Ultimately, one new clavatol derivate, penicophenone F (**1**), and seven known clavatols (**2**–**7**) were identified from the crude extract of *Penicillium* sp. MYA5 after 45 days of PDA growth.

### 2.2. Structural Characterization of Compounds ***1**–**7***

**Figure 5:**
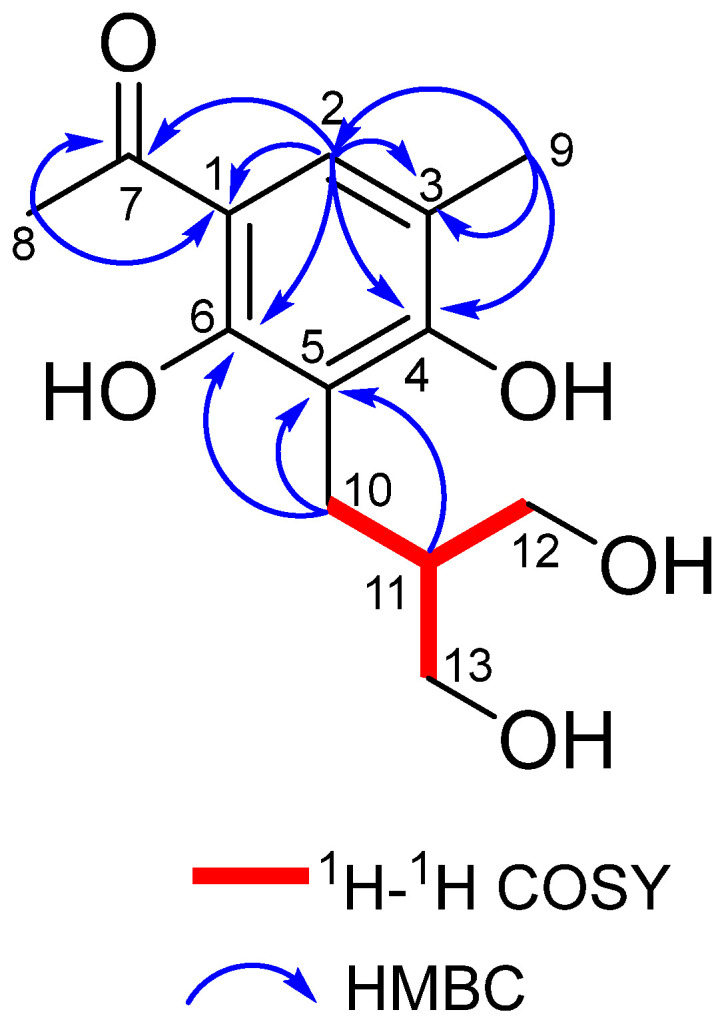
COSY and HMBC correlations of compound **1**.

Compound **1** was isolated as a yellow amorphous solid. Its molecular formula was determined to be C_13_H_18_O_5_ by HRESIMS (*m/z* 277.1048 [M + Na]^+^, calculated for C_13_H_18_O_5_Na 277.1046), indicating five degrees of unsaturation. The IR spectrum exhibited major absorption bands at 3331 cm^−1^, 2954 cm^−1^, 2922 cm^−1^, 2852 cm^−1^, and 1449 cm^−1^, corresponding to hydroxyl and aromatic rings, respectively [19]. The ^1^H NMR spectrum of **1** revealed signals attributed to two methyl singlets at *δ*_H_ 2.18 and 2.55 and one aromatic proton singlet at *δ*_H_ 7.52 (Table 1). The ^13^C NMR spectrum of **1** revealed one ketone carbonyl at *δ*_C_ 203.2, two oxygenated quaternary carbons at *δ*_C_ 161.1 and 161.5, three sp^2^ hybridized carbons at *δ*_C_ 112.4, 112.9, and 116.7, and two oxygenated methylenes at *δ*_C_ 61.8 and 61.8 (Table 1). A comparison of the ^1^H and ^13^C NMR data of **1** with those of the known compound **2** revealed that they shared the same phenol skeleton [20], which was further confirmed by the HMBC from H-9 to C-3, C-4, and C-2, from H-2 to C-7, C-1, C-6, C-4, and C-3, and from H-8 to C-1 and C-7, as shown in Figure 5. The COSY spectrum revealed the presence of one isolated spin system: C-10–C-11–C-12–C-13 (Figure 5). HMBC correlations from H-11 to C-5, from H-10 to C-5, and from H-10 to C-6 provided evidence of the direct linkage between C-10 and C-5. Furthermore, two hydroxy groups were attached to C-6 (δ_C_ 161.1) and C-4 (δ_C_ 161.5) based on the molecular formula, respectively.

Compounds **2**–**7** isolated in this study were identified as known compounds by comparing their NMR data and optical rotation values with the literature. These compounds included 1-(2,4-dihydroxy-5-methylphenyl) ethan-1-one (**2**) [20], communol G (**3**) [21], penicophenone C (**4**) [16], penicophenone A (**5**) [16], penicophenone E (**6**) [22], and penicophenone D (**7**) [16].

### 2.3. Biological Activity Assay

Compounds **1**–**7** were evaluated for antibacterial activity against *Staphylococcus aureus*, *Pseudomonas aeruginosa*, *Escherichia coli*, *Enterococcus faecalis*, *Vibrio vulnificus*, and *Vibrio parahaemolyticus* (Table 2). Compound **6** exhibited antibacterial activity against *S. aureus* and *E. coli* with MIC values of 4.0 μg/mL for both, whereas compounds **1**–**5** and **7** exhibited different levels of antibacterial activity against different microbes, as shown in Table 2.

In the current study, an in vitro ALR2 inhibition assay demonstrated that compounds **1** and **2**, with inhibition rates of 75.3% and 71.6% at a concentration of 10 μM, respectively, displayed superior inhibitory activities compared with other compounds (Table 3) with epalrestat as the positive control. Compounds **1** and **2,** therefore, represented the potential drug candidates for the treatment of diabetic complications.

**Figure 6:**
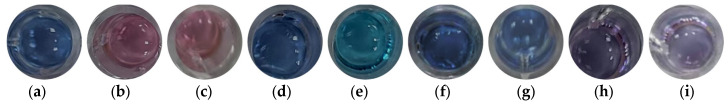
CAS assay results for compounds **1**–**7**, saturated EDTA solution, and blank control. (**a**) blank control, (**b**) saturated EDTA solution, (**c**) compound **1,** (**d**) compound **2,** (**e**) compound **3,** (**f**) compound **4,** (**g**) compound **5,** (**h**) compound **6,** (**i**) compound **7**.

The Fe(III)-binding properties of compounds **1**–**7** were assessed by the chrome azurol S (CAS) assay. Compounds **1**, **5**, and **6** showed binding activity (Table 4). Compounds **6** and **7** displayed a purple color when mixed with the CAS assay solution (Figure 6h,i), and compound **1** displayed a red color when mixed with the CAS assay solution (Figure 6c). Structurally, the compounds **1**, **5**, and **6** belong to the phenolates family. For this type of siderophore, the phenolic moiety and the number of this group are both very important in the interaction of these compounds with iron [23].

## 3. Materials and Methods

### 3.1. General Experimental Procedures

HPLC was conducted using a YMC-Pack Pro C18 RS (5 µm) column (YMC Co., Ltd., Kyoto, Japan) coupled with a Waters 1525 separation module (Waters Corp., Milford, MA, USA) equipped with a Waters 2998 photodiode array (PDA) detector (Waters Corp., Milford, MA, USA). High-resolution mass (ESI-HRMS) spectra were acquired using an Agilent Q-Tof micro YA019 (Agilent Technologies Inc., Lake Forest, CA, USA). NMR spectra were measured using Bruker AMX-500 instruments (Bruker Biospin Corp., Billerica, MA, USA) operating at 400 MHz for ^1^H and 100 MHz for ^13^C, with TMS as the internal standard. UV spectra were collected on a UV-8000 spectrophotometer (Shanghai Metash Instruments Co., Shanghai, China). IR(KBr). The spectra were recorded using a Jasco FTIR–400 spectrometer (Jasco Inc., Tokyo, Japan).

CD spectra were obtained using a Jasco J-715 spectropolarimeter (Jasco Inc., Tokyo, Japan). Optical rotations were determined using a Perkin-Elmer model 341 polarimeter (Perkin-Elmer Inc., Waltham, MA, USA). Silica gel (200–300 mesh, Qingdao Ocean Chemical Co., Qingdao, China), Sephadex LH-20 (18–110 µm, Pharmacia Co., Piscataway, NJ, USA), and ODS (50 µm, YMC Co., Ltd., Kyoto, Japan) were utilized for column chromatography (CC).

TLC analyses were conducted on pre-coated silica gel GF254 plates, and spots were visualized under UV light (254 nm) or by heating after spraying with anisaldehyde-H_2_SO_4_ reagent.

### 3.2. Biological Material and Bioinformatics Analysis

The strain *Penicillium* sp. MYA5 was isolated from the Arctic Svalbard Islands (E 10°35°, N 74°81°). The rDNA-ITS sequence of the strain was sequenced, and an ITS (SUB14112691) phylogenetic tree was constructed to identify the fungal strain as *Penicillium* sp. The strain has been deposited at the Chinese Typical Culture Preservation Center (CCTCC NO: SF2021062) and the Laboratory of Marine Biomedicine and Polar Medicine, Naval Specialty Medical Center, PLA Naval Medical University, Shanghai, People’s Republic of China.

The fungal strain underwent genome sequencing using the Illumina Hiseq 2000 platform at Shanghai Meiji Biomedical Technology, and its genomic data were stored at NCBI (JAYRCP010000000). To analyze the presence of biosynthetic gene clusters on the scaffolds, we employed antiSMASH https://fungismash.secondarymetabolites.org/#!/start (accessed on 15 May 2023) and 2ndFind https://biosyn.nih.go.jp/2ndfind/ (accessed on 16 May 2023). Gene prediction for fungi was performed using Maker v2.31.9, while rRNAs and tRNAs contained in the genome were predicted using Barrnap v0.8 and tRNAscan-SE v2.0, respectively. The gene annotation results were used to analyze the expression differences and gene ontology (GO) https://www.blast2go.com/ (accessed on 19 August 2023) and predict the protein-coding frames (CDS). GO enrichment analyses were performed. Verification was also conducted by Blast analysis https://blast.ncbi.nlm.nih.gov/Blast.cgi (accessed on 23 September 2023) by manually comparing the homologous gene/protein sequences in the GenBank database.

### 3.3. Fermentation, Extraction, and Isolation

The fungus *Penicillium* sp. MYA5 was cultured on potato dextrose agar medium at 28 °C for 45 days. After cultivation, the culture underwent extraction by immersion in a methanol–dichloromethane mixture (1:1) for 24 h, followed by ultrasonication for 30 min to disrupt the cells. The resulting mixture was filtered through eight layers of gauze to obtain the filtrate. Subsequently, the culture underwent three rounds of ultrasonic extraction, and the combined filtrates were concentrated under reduced pressure using a rotary evaporator until all the organic solvents were removed. An appropriate amount of water suspension was added, followed by three additional extractions with an equal volume of ethyl acetate. The ethyl acetate layer was collected and further concentrated under reduced pressure using a rotary evaporator until dry, yielding 17.38 g of the fermented extract. The total fermented extract was then subjected to silica gel (200–300 mesh) column chromatography and separated into nine fractions (Fr. A–Fr. I) using a step gradient elution of petroleum ether and ethyl acetate.

Fr. F was fractionated using ODS-C18 column chromatography (MeOH/H_2_O, 3:7 → 10:0), yielding eight sub-fractions (Fr. F1–Fr. F8). The purification of the Fr. F2 fractions via semi-preparative HPLC (acetonitrile–water, 50:50 *v*/*v*, flow rate: 2 mL/min) resulted in the isolation of compounds **3** (3.0 mg, retention time: 11 min, λ = 219 nm) and **2** (4.0 mg, retention time: 14 min, λ = 218 nm).

Fr. H underwent fractionation using ODS-C18 column chromatography (MeOH/H_2_O, 3:7 → 10:0), yielding ten sub-fractions (Fr. H1–Fr. H10). The purification of Fr. H3 via semi-preparative HPLC (methanol–water, 50:50 *v*/*v*, flow rate: 2 mL/min) led to the isolation of compound **6** (6.3 mg, retention time: 47 min, λ = 220 nm). Similarly, the purification of Fr. H9 by semi-preparative HPLC (acetonitrile–water, 50:50 *v*/*v*, flow rate: 2 mL/min) resulted in the isolation of compound **5** (1.5 mg, retention time: 33 min, λ = 203 nm).

Fr. I was fractionated using ODS-C18 column chromatography (MeOH/H_2_O, 4:6 → 10:0), producing eleven sub-fractions (Fr. I1–Fr. I11). Normal-phase silica gel column chromatography was performed on Fr. I4 with a petroleum ether-methanol gradient (100:1–1:1), yielding seven components (Fr. I4a–Fr. I4g) based on TLC chromatography. Fr. I4a was further purified via semi-preparative HPLC (acetonitrile-water, 60:40 *v*/*v*, flow rate: 2 mL/min), resulting in the isolation of compound **7** (2.9 mg, retention time: 34 min, λ = 219 nm).

Fraction J was fractionated using ODS-C18 column chromatography (MeOH/H_2_O, 4:6 → 10:0), resulting in eight sub-fractions (Fr. J1–Fr. J8). Fraction J3 was further separated via normal-phase silica gel column chromatography with a petroleum ether–methanol gradient (100:1–1:1), yielding five fractions (Fr. J3a–Fr. J3e). Subsequently, Fr. J3b underwent purification through semi-preparative HPLC (acetonitrile–water, 25:75 *v*/*v*, flow rate: 2 mL/min), leading to the isolation of compound **1** (2.5 mg, retention time: 52 min, λ = 218 nm). Fr. J6 was subjected to normal-phase silica gel column chromatography with a petroleum ether–methanol gradient (100:1–1:1), yielding six fractions (Fr. J6a–Fr. J6f). Fr. J6c was then purified via semi-preparative HPLC (acetonitrile–water, 60:40 *v*/*v*, flow rate: 2 mL/min), resulting in the isolation of compound **4** (2.3 mg, retention time: 35 min, λ = 218 nm).

Penicophenone F (**1**): yellow, amorphous solid; IR(KBr) *ν*_max_ 3331, 2954, 2922, 2852, 1737, 1621, 1449, 1421, 1370, 1331, 1272, 1229, 1187, 1082, 986, 822, 737, and 570 cm^−1^; ^1^H NMR (400 MHz) and ^13^C NMR (100 MHz) (Table 1); HR-ESIMS *m*/*z* 254.1046 [M + Na]^+^ (calculated for C_13_H_18_O_5_Na, 277.1046).

### 3.4. Analysis of Transcriptomic Data and RT-qPCR

*Penicillium* sp. MYA5 underwent culturing under two distinct conditions: potato dextrose agar (PDA) for 45 days and KSB solid medium (containing glucose 12.36 g/L, tryptone 1.05 g/L, beef extract 6.08 g/L, manganese sulfate monohydrate 0.246 g/L, agar 20 g/L) for 45 days. The fermented crude extract was then collected. Sequencing was conducted using the Illumina Novaseq 6000 platform by Shanghai Meiji Biomedical Technology. To validate the RNA-seq data, RT-qPCR was performed following established protocols [24]. The primers utilized for RT-qPCR analysis are detailed in Table S1, with the 16S rDNA–coding gene employed as the internal control.

### 3.5. In Vitro ALR2 Enzyme Inhibitory Activity Assay

100 µL of reaction mixture was composed of 20 µL of buffer (100 mM sodium phosphate, pH 6.2), 30 µL of enzyme extract, 20 µL of substrate (10 mM), 20 µL of cofactor (0.1 mM of NADPH), and 10 µL of test compound (1 mM) [25,26]. The reaction mixture without cofactor was incubated at 32 °C for 10 min, then the enzymatic reaction was initiated with the addition of NADPH and monitored for 5 min. DL-glyceraldehyde was used as the substrate for the ALR2 assays.

The compounds were dissolved in 100% DMSO and diluted with deionized water, keeping the DMSO concentration equal to 0.1% in the assay. The compounds were initially tested for percent inhibition at a concentration of 10 µM. ALR2 activity was determined by monitoring the change in absorbance at 340 nm caused by decreasing the NADPH [27]. Epalrestat served as the positive control.

$$\mathrm{Inhibition}\;\mathrm{rate}:\;\frac{\left[({OD}_{drug\;hole}-{OD}_{drug\;blank\;hole})-({OD}_{metabolism\;hole}-{OD}_{blank\;hole})\right]}{\left({OD}_{standard\;hole}-{OD}_{blank\;hole}\right)}\times 100\%$$

### 3.6. Antibacterial Assay

The antimicrobial activities of compounds **1**–**7** against *Staphylococcus aureus*, *Escherichia coli*, *Pseudomonas aeruginosa*, *Enterococcus faecalis*, *Vibrio vulnificus*, and *Vibrio parahaemolyticus* were assessed using the broth dilution method [28,29]. Levofloxacin hydrochloride served as the positive control.

### 3.7. Chrome Azurol S Assay

An assay solution was prepared to measure the iron-binding activity of the compounds [30]. Hexadecyltrimethylammonium bromide (CTAB) (21.9 mg) was diluted in 25 mL of H_2_O at 35 °C. An iron(III) chloride solution (1.5 mL, 1.0 mM) (prepared by dissolving FeCl_3_·6H_2_O in a 10 mM aqueous HCl solution) and 7.5 mL of a 2.0 mM aqueous chrome azurol S (CAS) solution were added to this solution. In a separate container, 9.76 g of 2-(N-morpholino) ethanesulfonic acid (MES) was dissolved in 50 mL of H_2_O, and the pH of this solution was adjusted to 5.6 with a 50% KOH solution. Then, the previous CTAB-CAS-Fe(III) solution was mixed with the MES buffer solution slowly, and we filled the solution with water to obtain 100 mL. The modified CAS assay solution (100 μL) was loaded into each well of a 96-well plate, which was then mixed with 100 μL of solution of compounds diluted in H_2_O. The final concentrations of each compound were 10 μg/mL. The color changes were observed by visual inspection after incubation at 37 °C for 1 h. Then, the corresponding absorbance changes were measured on a microplate reader at 630 nm. The saturated EDTA solution (which reacts with CAS detection solution in red) was used as the positive control, and the solvent dissolving the sample was mixed with CAS detection solution in the same volume as the blank control. The color changed to red, orange red, and purple, indicating that the compounds could bind with iron.

## 4. Conclusions and Discussion

In summary, genomic and transcriptomic approaches were employed to activate the expression of the *A5cla* cluster in *Penicillium* sp. MYA5. One new clavatol derivate, penicophenone F (**1**), and seven known clavatols (**2**–**7**) were isolated, and the structures of all the compounds were elucidated through comprehensive spectroscopic analysis. The moderate inhibitory activities of compounds **1** and **2** against ALR2 enzyme were demonstrated. Compounds **1**, **5**, and **6** showed potential antibiotic activity and iron-binding ability.

Siderophores produced by endophytic fungi played a pivotal role in supporting plant growth. It was conjectured that clavatols extracted from *Penicillium* sp. MYA5 might similarly enhance the survival and development of *Dryas octopetala* in iron-deficient polar environments. The synthetic biogenesis cluster of hydroxylate-type siderophores was annotated in *Penicillium* sp. MYA5. Hydroxamate-type siderophores constituted the primary class of siderophores synthesized by endophytic fungi [31]. The correlation analysis of the *A5cla* cluster and the hydroxylate-type siderophore biosynthesis cluster revealed that the genes contained in the two clusters were significantly correlated (Figure S12). It was hypothesized that clavatols might act synergistically with other forms of siderophores to facilitate the normal growth and development of hosts in extreme environments. Overall, this work underscored the utility of integrating genomic and transcriptomic approaches as a systematic approach to expedite the discovery of bioactive compounds.

## Figures and Tables

**Table 1:** ^1^H (400 MHz) and ^13^C NMR (100 MHz) spectroscopic data of **1** in CDCl_3_.

Position	*δ*_C_	δ_H_, Mult. (*J* in Hz)
1	112.4, C	
2	130.5, CH	7.52, s
3	116.7, C	
4	161.5, C	
5	112.9, C	
6	161.1, C	
7	203.2, C	
8	24.9, C	2.55, s
9	14.9, CH_3_	2.18, s
10	20.5, CH_2_	2.70, d (6.9)
11	42.5, C	2.00, m
12	61.8, CH_2_	3.57, dd (10.8, 6.3)/3.62, dd (10.8, 5.1)
13	61.8, CH_2_	3.57, dd (10.8, 6.3)/3.62, dd (10.8, 5.1)

**Table 2:** Antibacterial activity of compounds **1**–**7**.

Compounds	MIC (μg/mL)					
	*S. aureus*	*E. coli*	*P. aeruginosa*	*V. vulnificus*	*E. faecalis*	*V. parahaemolyticus*
**1**	NA	32.0	NA	NA	NA	NA
**2**	32.0	NA	NA	NA	32.0	32.0
**3**	NA	NA	NA	NA	NA	NA
**4**	16.0	32.0	NA	32	32.0	32.0
**5**	16.0	16.0	NA	32.0	32.0	NA
**6**	4.0	4.0	16.0	NA	32.0	NA
**7**	32.0	32.0	NA	NA	NA	NA
levofloxacin hydrochloride ^a^	2.0	1.0	1.0	1.0	1.0	1.0

^a^ Positive control. ‘NA’ stands for no inhibitory effect at greater than 64 μg/mL.

**Table 3:** Inhibition ratio of ALR2 of compounds **1**–**7**.

Compounds	Inhibition Ratio (%)
**1**	75.3
**2**	71.6
**3**	52.2
**4**	26.6
**5**	33.7
**6**	52.4
**7**	47.9
Epalrestat ^a^	96.5

^a^ Positive control.

**Table 4:** CAS assay results for compounds **1**–**7**, saturated EDTA solution, and blank control.

Compounds	OD_630_
**1**	0.093
**2**	0.262
**3**	0.395
**4**	0.239
**5**	0.250
**6**	0.115
**7**	0.198
saturated EDTA solution	0.053
blank control	0.254

## Data Availability

The data presented in this study are available upon request from the corresponding author.

## Supplementary Materials

The following supporting information can be downloaded at https://www.mdpi.com/article/10.3390/md22060236/s1, Table S1: Primers used in this study; Table S2: AntiSMASH’s annotation results; Table S3: Blastp alignment structure of cluster *3.1* (*A5cla*); Table S4: ^13^C NMR data for compounds **2**–**7**; Table S5: ^1^H NMR data for compounds **2**–**7**; Figure S1: Color reaction of *Penicillium* sp. MYA5 on CAS plating medium; Figure S2: Phylogenetic tree of the ITS sequence of *Penicillium* sp. MYA5; Figure S3: ^1^H NMR spectrum of penicophenone F (**1**) in CD_3_OD, 400 MHz; Figure S4: ^13^C NMR spectrum of penicophenone F (**1**) in CD_3_OD, 100 MHz; Figure S5: DEPT135 spectrum of penicophenone F (**1**) in CD_3_OD, 100 MHz; Figure S6: HSQC spectrum of penicophenone F (**1**) in CD_3_OD, 100 MHz; Figure S7: COSY spectrum of penicophenone F (**1**) in CD_3_OD, 100 MHz; Figure S8: HMBC spectrum of penicophenone F (**1**) in CD_3_OD, 100 MHz; Figure S9: NOESY spectrum of penicophenone F (**1**) in CD_3_OD, 100 MHz; Figure S10: HRESIMS of penicophenone F (**1**); Figure S11: IR spectrum of penicophenone F (**1**); Figure S12: Expression correlation analysis of siderophore biosynthesis related genes.
