# Supplementary material for: Genomics- and Transcriptomics-Guided Discovery of Clavatols from Arctic Fungi Penicillium sp. MYA5
Source: Mar Drugs. 2024 May 22;22(6):236. doi: 10.3390/md22060236 (PMC11205228; doi:10.3390/md22060236)

Supporting Information

Genomics- and Transcriptomics-Guided Discovery of Clavatols from Arctic Fungi *Penicillium* sp. MYA5

Yuan-Yuan Sun [1, †], Bo Hu [1, †], Hao-Bing Yu [1, †], Xian-Chao Meng [1], Zhe Ning [1], Jing Zhou [2], Jin-Feng Ding [1], Ming-Hui Cui [1] and Xiao-Yu Liu [1, *]

[1] Naval Medical Center of PLA, Department of Marine Biomedicine and Polar Medicine, Naval Medical University, Shanghai 200433, China; sunyy3636@163.com (Y.-Y.S.); hb8601@163.com (B.H.); yuhaobing1986@126.com (H.-B.Y.); Ningzhe95@163.com (Z.N.); Mxc0960623@163.com (X.-C.M.); 2022220806@jou.edu.cn (J.-F.D.); 13156270892@163.com (M.-H.C.)

[2] Institute of Quality Inspection and Technical Research, Shanghai 200031, China; zhoujing2@sqi.org.cn (J.Z.)

* Correspondence: biolxy@163.com; Tel.: +86-21-81883267

† These authors contributed equally to this work.

Content of Supporting Information

Table S1. Primers used in this study.

Table S2. AntiSMASH's annotation results.

Tabel S3. Blastp alignment structure of cluster *3.1* (*A5cla*).

Tabel S4. ^{13}C NMR data for compounds **2**–7.

Tabel S5. ^{1}H NMR data for compounds **2**–7.

Figure S1. Color reaction of *Penicillium* sp. MYA5 on CAS plating medium

Figure S2. Phylogenetic tree of the ITS sequence of *Penicillium* sp. MYA5.

Figure S3. ^{1}H NMR spectrum of penicophenone F (**1**) in CD_3OD, 400 MHz.

Figure S4. ^{13}C NMR spectrum of penicophenone F (**1**) in CD_3OD , 100 MHz.

Figure S5. DEPT135 spectrum of penicophenone F (**1**) in CD_3OD , 100 MHz.

Figure S6. HSQC spectrum of penicophenone F (**1**) in CD_3OD , 100 MHz.

Figure S7. COSY spectrum of penicophenone F (**1**) in CD_3OD , 100 MHz$_3$.

Figure S8. HMBC spectrum of penicophenone F (**1**) in CD_3OD , 100 MHz.

Figure S9. NOESY spectrum of penicophenone F (**1**) in CD_3OD , 100 MHz.

Figure S10. HRESIMS of penicophenone F (**1**).

Figure S11. IR spectrum of penicophenone F (**1**).

Figure S12. Expression correlation analysis of siderophore biosynthesis related genes.

Table S1. Primers used in this study.

Primer name	base sequence (5′–3′)
3818-F	CCAGCAAGGTCGCCAATGTG
3818-R	GAGGGAAACTCGTCGTGATAAGC
3819-F	TCAGCAGCAGAAGCATACAATACC
3819-R	ATGTCGGCGATGTGGATAGAGG
3820-F	CCGATGCTGGACCTATTGCTATTC
3820-R	ACTCGTAGGCGGCGTTCTC
3821-F	ATGTGGTTCTGTCGCTTCTTGC
3821-R	GCTTCTTTCCGTCCTGTTTCTCC
3822-F	TGGATTGCGAGACAAGGGACTAG
3822-R	TCTGGTGAATAACAAGCGAGACATC
3823-F	GTAATGGCGGTGCGGATTGC
3823-R	CTTGAGAACCTGCGACGACTTG
3824-F	GTAATGGCGGTGCGGATTGC
3824-R	CTTGAGAACCTGCGACGACTTG
3825-F	AACAGTGGCGGAACCTAGATAGAG
3825-R	ATCCTTGCCTTCTTACACGATTGG
3826-F	CATCAGCAAAGAAGACGCAATCATC
3826-R	CAACCGCCATCATTCCTACAGAG
3827-F	CATCAGCAAAGAAGACGCAATCATC
3827-R	CAACCGCCATCATTCCTACAGAG
3828-F	GGCTGTTCCGTTACCTATTCTTCTG
3828-R	TTCCCTCCTGTTTCTCGACTATACC
A516S-F	TCCGCCGCAAGATTCTCAATTC
A516S-R	GCCTCGTCCTCGCTTCCTC

Table S2. AntiSMASH's annotation results.

Cluster	Type	Similarity to known BDC	Cluster	Type	Similarity to known BDC	Cluster	Type	Similarity to known BDC
Cluster1.1	NRPS-like		*Cluster3.2*	NRPS		*Cluster6.3*	NRPS、T1-PKS	
Cluster1.2	NRPS-like		*Cluster3.3*	NRPS-like、TI-PKS		*Cluster7.1*	T1-PKS、NRPS	100% AbT1
Cluster1.3	NRPS-like		*Cluster3.4*	NRPS		*Cluster7.2*	NRPS	
Cluster1.4	NRPS		*Cluster3.5*	T1-PKS、NRPS		*Cluster8.1*	Terpene	
Cluster1.5	T1-PKS		*Cluster4.1*	T1-PKS、NRPS		*Cluster8.2*	NRPS-like	
Cluster2.1	NRPS	21% penigequinolone	*Cluster4.2*	T1-PKS	25% pyrioyropene	*Cluster9.1*	T1-PKS、NRPS	100% dimethylcoprogen
Cluster2.2	Terpene		*Cluster4.3*	T1-PKS		*Cluster9.2*	T1-PKS	
Cluster2.3	NRPS		*Cluster4.4*	NRPS-like		*Cluster9.3*	NRPS、T1-PKS	
Cluster2.4	T1-PKS、NRPS-like		*Cluster5.1*	NRPS-like、TI-PKS	22% viridicatumtoxin	*Cluster10.1*	terpene	60% squalestatin
Cluster2.5	NRPS、	18%	*Cluster5.2*	T1-PKS	100%	*Cluster11.*	T1-PKS、	26%

	indole、terpene	shearinine D			naphthopyrone	*1*	NRPS-like	neurosporin A
Cluster2.6	Indole	40% penitres	*Cluster5.3*	NRPS、indole	42% histidyltryptophany -ldiketopiperazine	*Cluster11.2*	NPRS	
Cluster2.9	NRPS-like		*Cluster5.4*	terpene		*Cluster14.1*	NRPS-like	
Cluster2.10	betalactone		*Cluster6.1*	NRPS-like		*Cluster17.1*	NRPS	75% nidulanin
Cluster3.1	T1-PKS	12% citrinin	*Cluster6.2*	T1-PKS	100% melanin	*Cluster18.1*	NRPS	

Table S3. Blastp alignment structure of cluster *3.1* (*A5cla*).

Gene	Gene name	Homologous gene	Homologous strain	Protein	Identity (%)	Coverage (%)
gene03818	*A5claA*	A0A481WNM7.1	*Penicillium crustosum*	TF claA	100	98
gene03819	*A5claB*	XP_056728458.1	*Penicillium crustosum*	hypothetical protein	100	95
gene03820	*A5claC*	A0A481WNM8.1	*Penicillium crustosum*	Enoyl-CoA enoyl-CoA hydratase/Isomerase claC	100	99
gene03821	*A5claD*	A0A481WNL2.1	*Penicillium crustosum*	Fe(II) oxygenase claD	100	98
gene03822	*A5claE*	XP_056728455.1	*Penicillium crustosum*	hypothetical protein	100	97
gene03823	*A5claF*	A0A481WQB6.1	*Penicillium crustosum*	NR-PKS claF	97.48	100
gene03824	*A5claG*	A0A481WQ01.1	*Penicillium crustosum*	ABC-type transporter claG	89.13	58
gene03825	*A5claH*	EKV04078.1	*Penicillium digitatum* PHI26	hydrolase claH	66.55	98

gene03826	*A5claI*	A0A481WR96.1	*Penicillium crustosum*	HR-PKS claI	99.25	99
gene03827		A0A481WR96.1	*Penicillium crustosum*	HR-PKS claI	100	85
gene03828	*A5claJ*	A0A481WPJ6.1	*Penicillium crustosum*	Cytochrome P450 monooxygenase claJ	100	93

Table S4. ^{13}C NMR data for compounds **2**–7.

Carbon	2	3	4	5	6	7
1	118.5, C	117.6, C	161.07, C	155.74, C	13.0, CH_3	160.2, C
2	164.5, C	167.6, C	118.41, C	116.23, C	15.4, CH_3	119.5, C
3	103.0, CH	122.4, C	131.2, CH	128.66, CH	26.3, CH_3	130.68, CH
4	165.1, C	165.5, C	112.78, C	112.06, C	81.0, CH_2	113.28, C
5	114.2, C	123.1, C	161.85, C	159.22, C	98.8, C	158.76, C
6	135.6, CH	134.4, CH	110.6, C	109.85, C	106.0, C	112.83, C
7	204.4 C	54.1, CH_2	22.0, CH_2	22.14, CH_2	114.1, C	14.5, CH_2
8	26.5, CH_3	205.8, C	143.39, C	30.64, CH	117.5, C	101.46, C
9	15.8, CH_3	26.9, CH_3	191.3, C	103.25, C	130.8, CH	177.19, C
10	-	16.0, CH_3	116.8, C	46.67, CH	155.3, C	76.17, CH
11	-	62.5, CH_3	151.69, C	206.82, C	159.9, C	175.8, C
12	-	-	183.72, C	44.87, CH_2	173.7, C	15.91, CH_3
13	-	-	139.82, C	67.50, CH	203.3, C	203.5, C
14	-	-	16.18, CH_3	14.37, CH_3	-	26.03, CH_3
15	-	-	202.8	202.11, C	-	17.25, CH_3
16	-	-	26.3, CH_3	25.52, CH_3	-	-
17	-	-	8.18, CH_3	14.83, CH_3	-	-

18	-	-	12.5, CH_3	7.06, CH_3	-	-
19	-	-	-	21.38, CH_3	-	-

Table S5. ^{1}H NMR data for compounds **2**–7.

Position	2	3	4	5	6	7
1	-	-	-	-	1.52, (s)	-
2	-	-	-	-	2.18, (s)	-
3	7.55, (s)	-	7.32 (s)	7.27 (s)	2.52 (s)	7.41 (s)
4	-	-	-	-	4.87 (s)	-
5	-	-	-	-	-	-
6	6.23, (s)	7.72, s	-	-	-	-
7	-	4.68, s	3.75 (s)	2.57 (m) 2.77 (dd, 16.5, 5.5)	-	3.45 (q, J = 15.2 Hz)
8	2.51 (s)	-	-	2.0 (m)	-	-
9	2.11 (s)	2.59, s	-	-	7.37 (s)	-
10	-	2.25, s	-	2.99 (q, 6.8)	-	4.85 (q, J = 6.8 Hz)
11	-	3.80, s	-	-	-	-
12	-	-	-	- 2.52 (d,12.7)	-	2.21, s
13	-	-	-	4.42 ,m	-	-
14	-	-	2.23, (s)	2.02 (s)	-	2.58, s
15	-	-	-	-	-	1.47, (d, J = 6.8 Hz)
16	-	-	2.54 (s)	2.54 (s)	-	-
17	-	-	1.92 (s)	1.20 (d,6.8)	-	-
18	-	-	2.38 (s)	1.15 (d, 7.6)	-	-
19	-	-	-	1.17 (d, 6.4)	-	-

Figure S1. Color reaction of *Penicillium* sp. MYA5 on CAS plating medium.

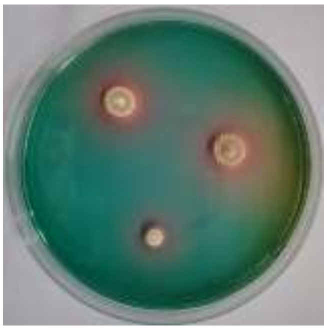

Figure S2. Phylogenetic tree of the ITS sequence of *Penicillium* sp. MYA5.

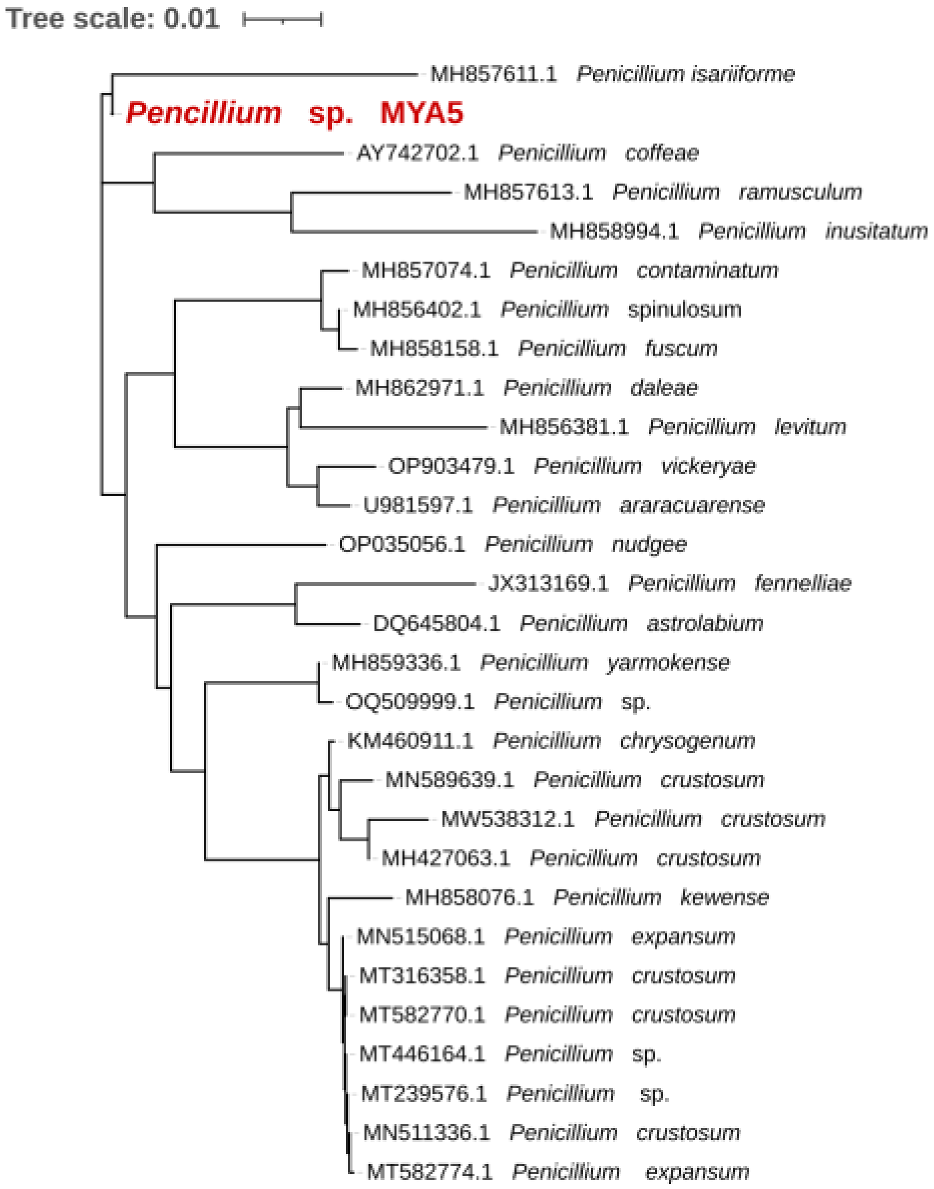

Figure S3. ^{1}H NMR spectrum of penicophenone F (**1**) in CD$_3$OD, 400 MHz.

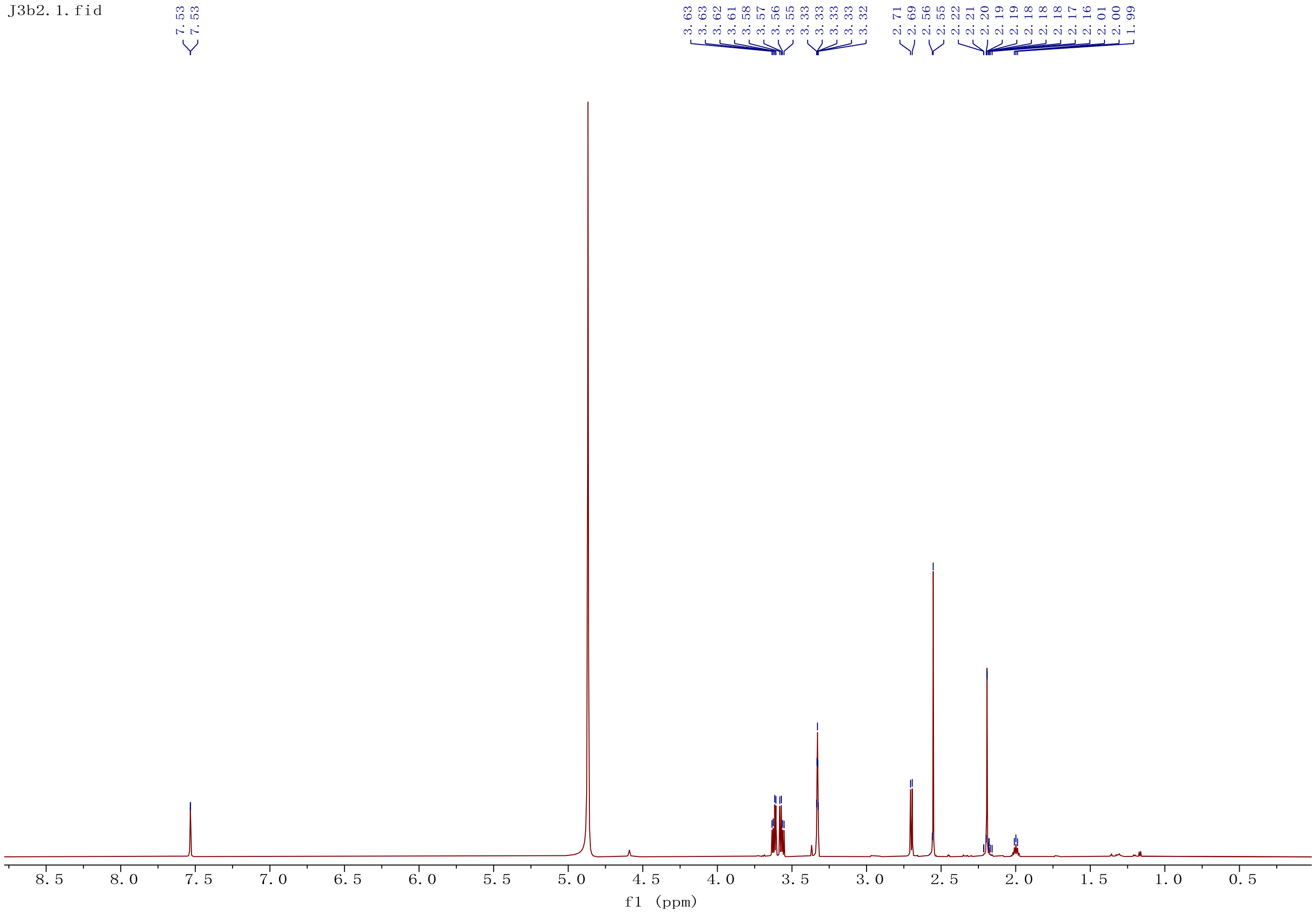

Figure S4. ^{13}C NMR spectrum of penicophenone F (**1**) in CD_3OD, 100 MHz.

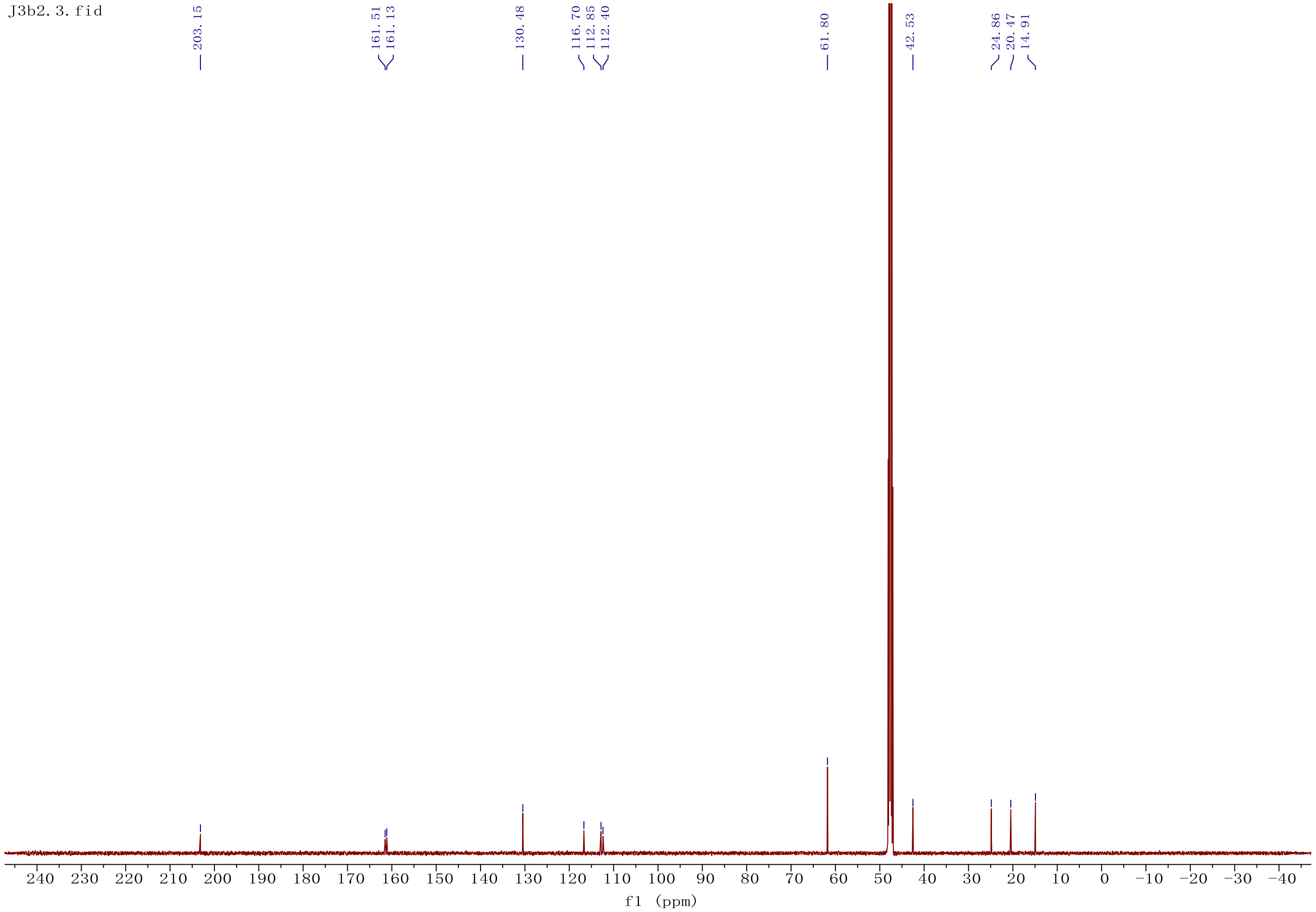

Figure S5. DEPT135 spectrum of penicophenone F (**1**) in CD_3OD, 100 MHz.

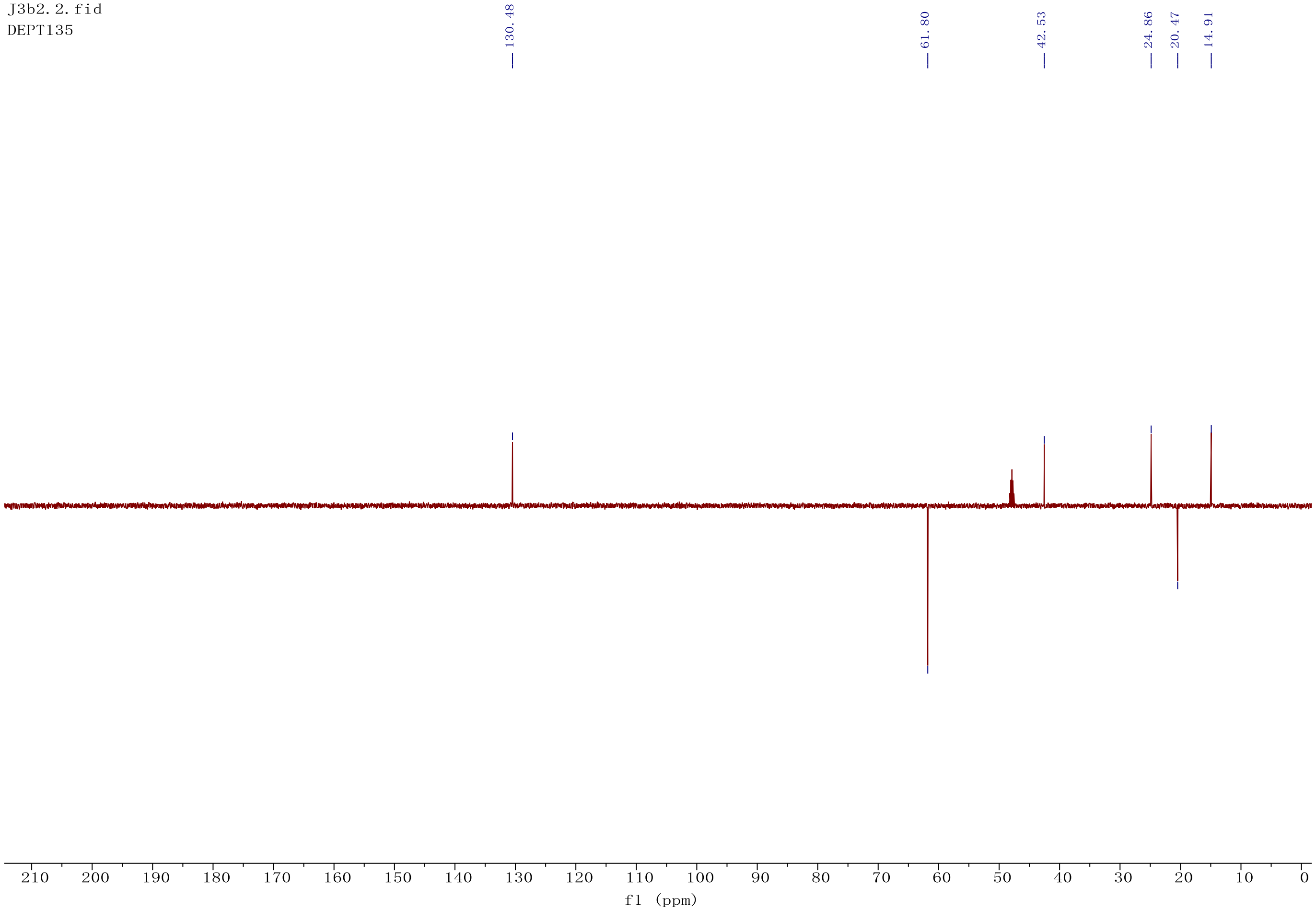

Figure S6. HSQC spectrum of penicophenone F (**1**) in CD_3OD, 100 MHz.

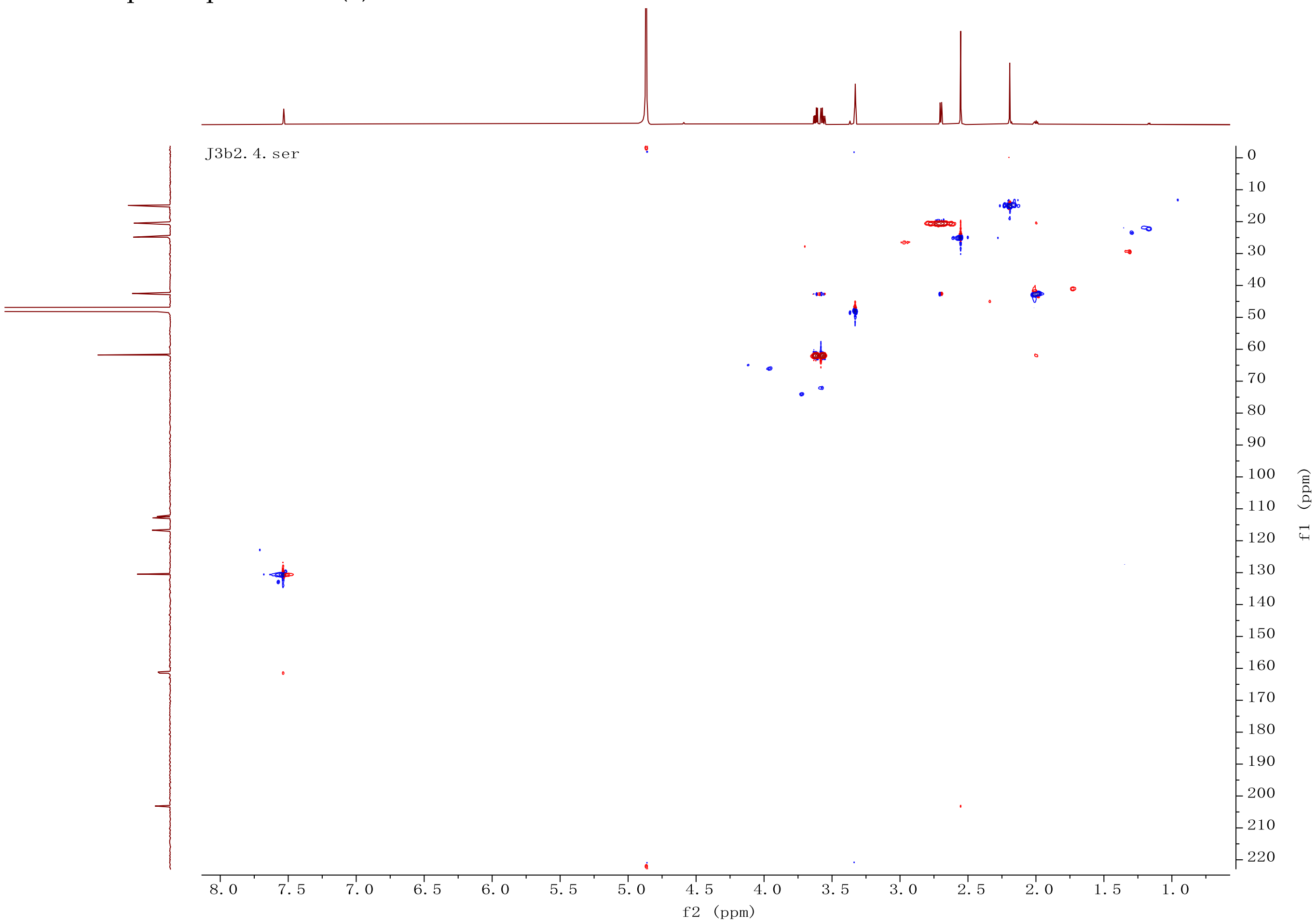

Figure S7. COSY spectrum of penicophenone F (**1**) in CD_3OD, 100 MHz.

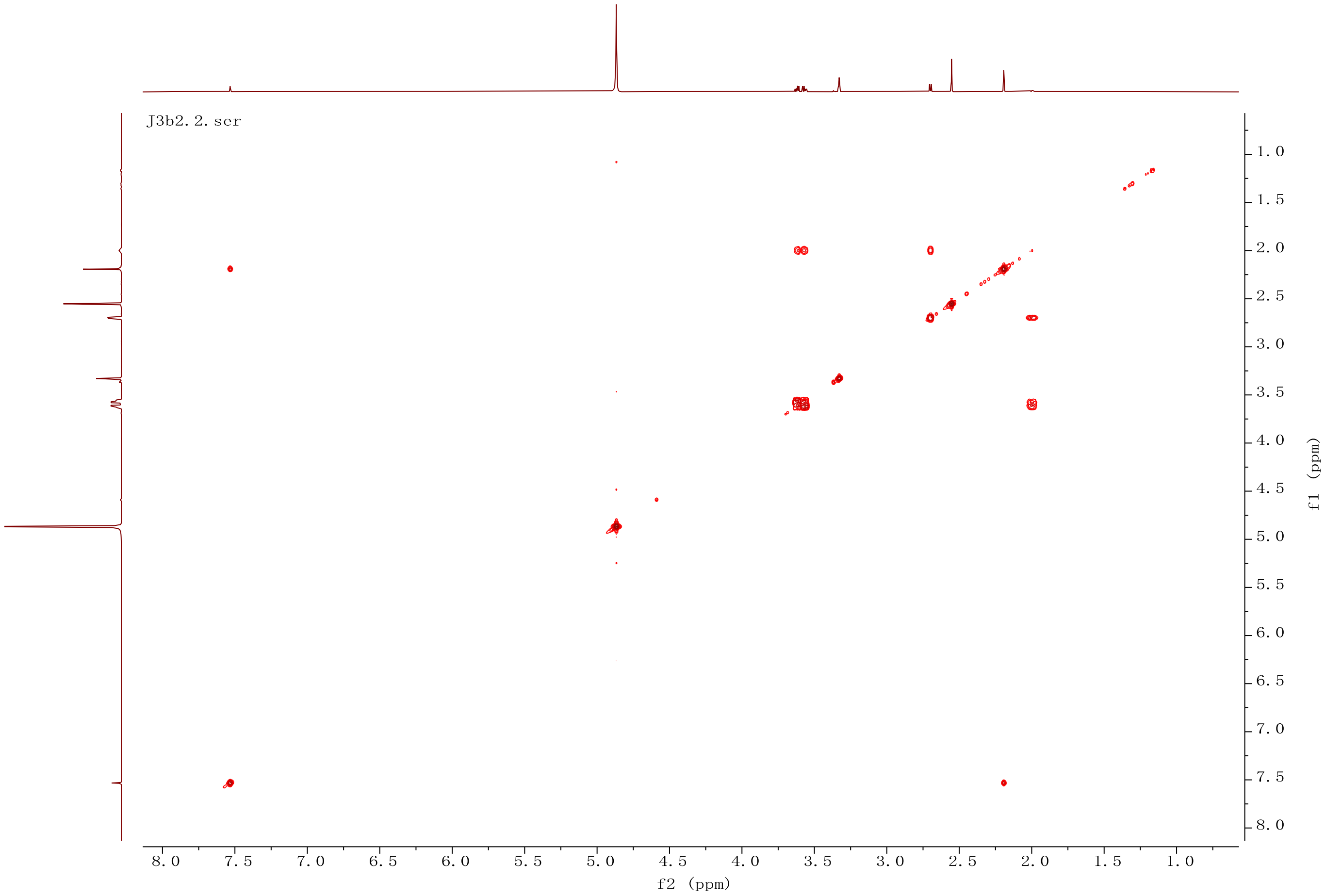

Figure S8. HMBC spectrum of penicophenone F (**1**) in CD_3OD, 100 MHz.

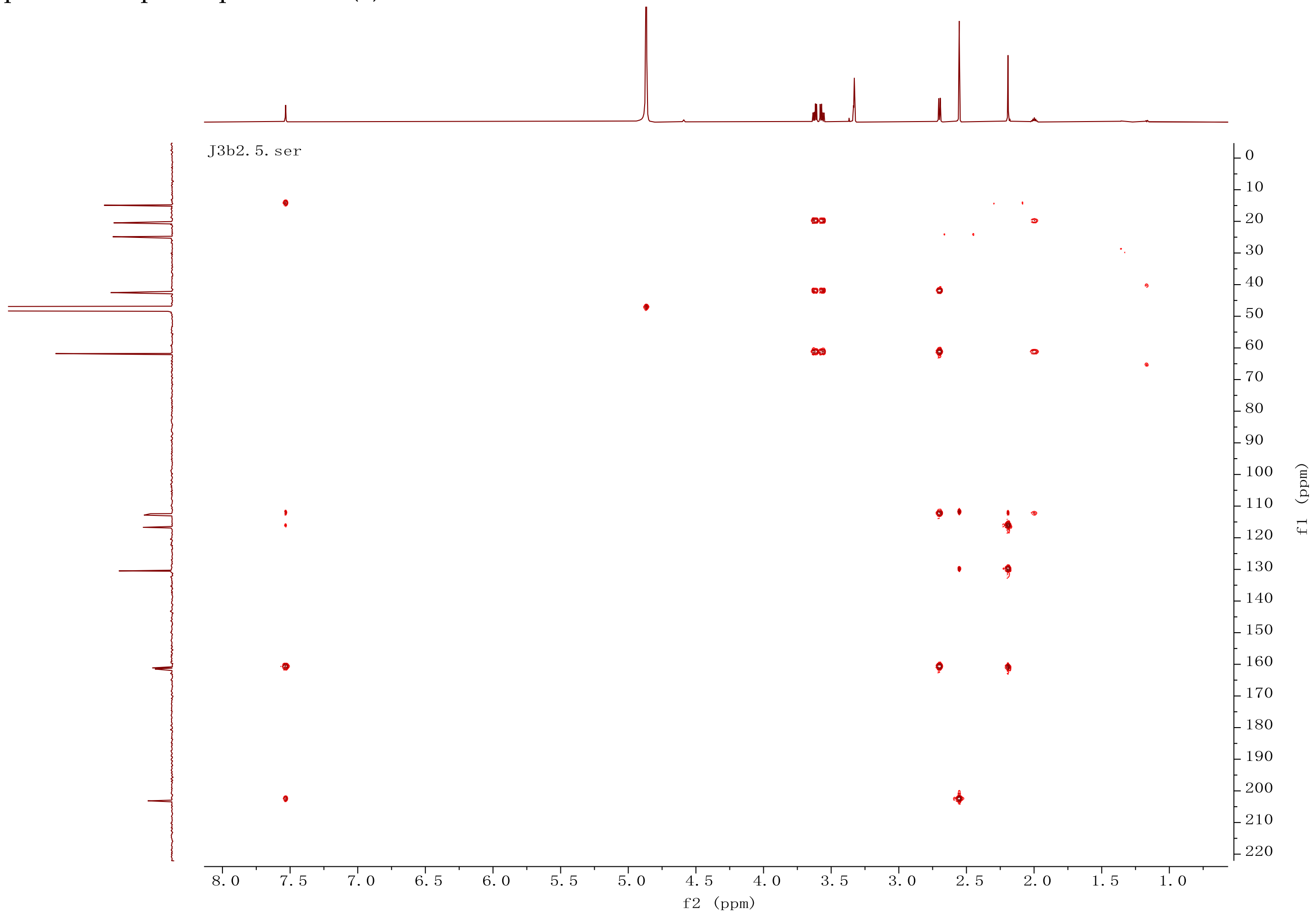

Figure S9. NOESY spectrum of penicophenone F (**1**) inCD3OD, 100 MHz.

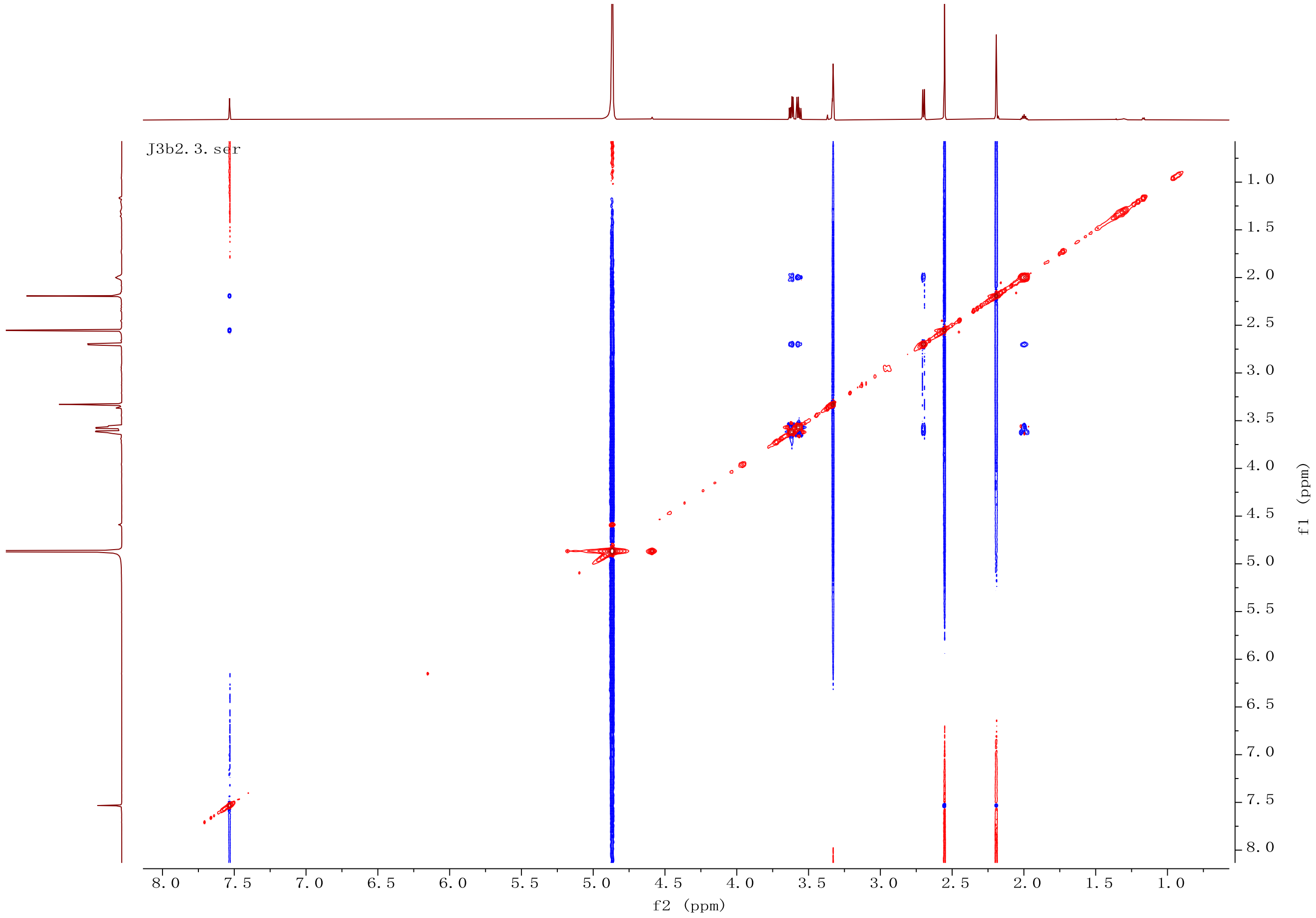

Figure S10. HRESIMS of penicophenone F (**1**).

Spectrum

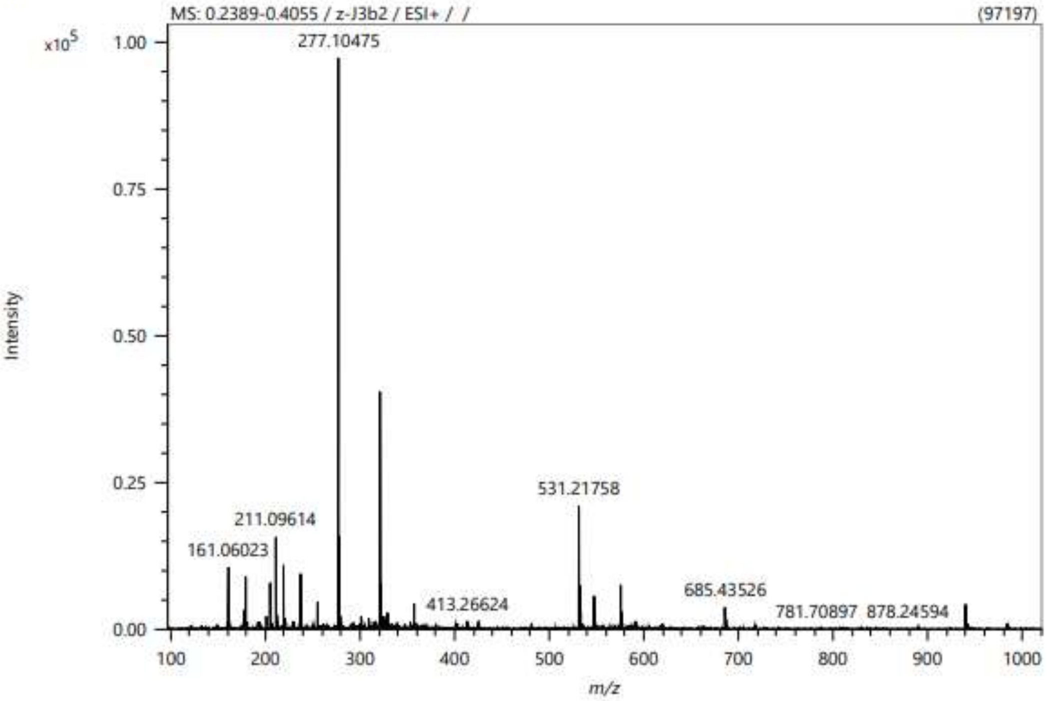

Elemental Composition

Parameters

Tolerance:	±5.00 ppm
Electron:	Odd/Even
Charge:	+1
DBE:	-1.5 - 200.0

Elements Set 1:

Symbol	C	H	N	O	Na	S	Cl	Br
Min	0	0	0	0	1	0	0	0
Max	200	200	0	10	1	0	0	0

Symbol	Si	F	B
Min	0	0	0
Max	0	0	0

Results

Mass	Intensity	Intensity [%]	Formula	Calculated Mass	Mass Difference [mDa]	Mass Difference [ppm]	DBE
277.10475	97197.32	100.00	C13 H18 O5 Na	277.10464	0.10	0.37	4.5

Figure S11. IR spectrum of penicophenone F (**1**).

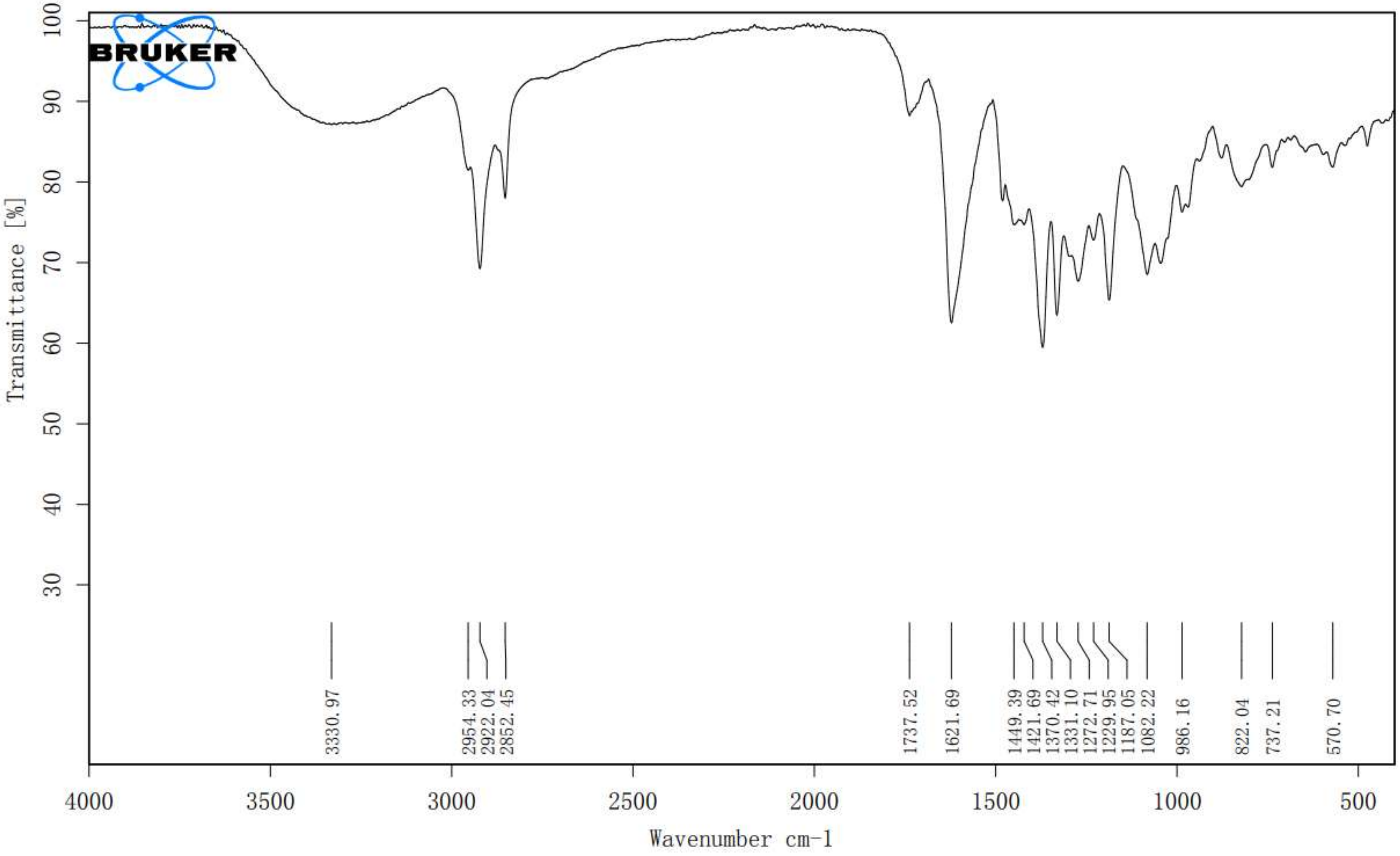

Figure S12. Expression correlation analysis of siderophore biosynthesis related genes.

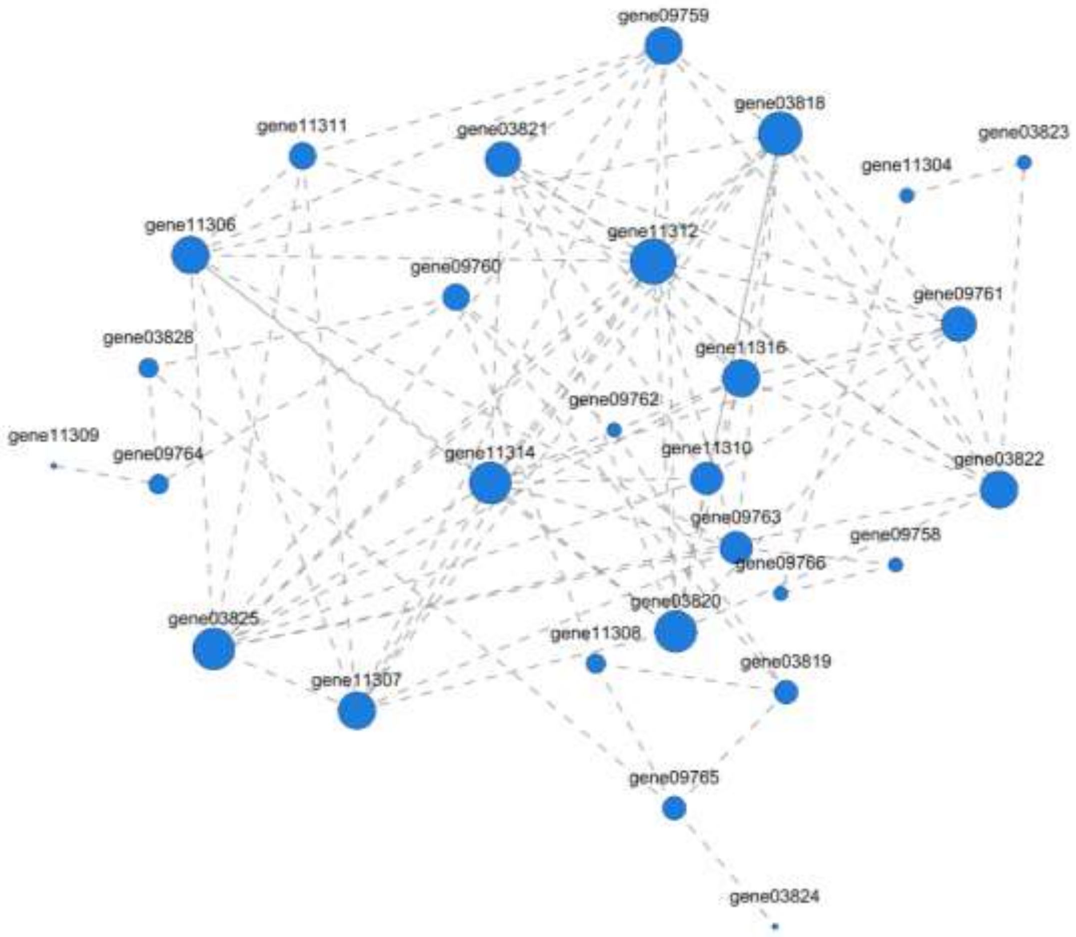

Supplement: Supplementary file 1 [file marinedrugs-22-00236-s001.zip › marinedrugs-2980375-supplementary.pdf]
